# Biphasic toxicodynamic features of some antimicrobial agents on microbial growth: a dynamic mathematical model and its implications on hormesis

**DOI:** 10.1186/1471-2180-10-220

**Published:** 2010-08-19

**Authors:** Miguel A Murado, José A Vázquez

**Affiliations:** 1 Grupo de Reciclado y Valorización de Materiales Residuales (REVAL) Instituto de Investigacións Mariñas (CSIC) r/Eduardo Cabello, 6. Vigo-36208. Galicia - Spain

## Abstract

**Background:**

In the present work, we describe a group of anomalous dose-response (DR) profiles and develop a dynamic model that is able to explain them. Responses were obtained from conventional assays of three antimicrobial agents (nisin, pediocin and phenol) against two microorganisms (*Carnobacterium piscicola *and *Leuconostoc mesenteroides*).

**Results:**

Some of these anomalous profiles show biphasic trends which are usually attributed to hormetic responses. But they can also be explained as the result of the time-course of the response from a microbial population with a bimodal distribution of sensitivity to an effector, and there is evidence suggesting this last origin. In light of interest in the hormetic phenomenology and the possibility of confusing it with other phenomena, especially in the bioassay of complex materials we try to define some criteria which allow us to distinguish between *sensu stricto *hormesis and biphasic responses due to other causes. Finally, we discuss some problems concerning the metric of the dose in connection with the exposure time, and we make a cautionary suggestion about the use of bacteriocins as antimicrobial agents.

**Conclusions:**

The mathematical model proposed, which combines the basis of DR theory with microbial growth kinetics, can generate and explain all types of anomalous experimental profiles. These profiles could also be described in a simpler way by means of bisigmoidal equations. Such equations could be successfully used in a microbiology and toxicology context to discriminate between hormesis and other biphasic phenomena.

## Background

The basic profile of dose-response (DR) relationships is a logical consequence of the population level required by this type of analysis. If the sensitivity of a population to an effector follows a unimodal distribution, then the profile of the corresponding cumulative function (i.e. the DR curve) will necessarily be a sigmoid. In practice, however, it is possible to find occasional anomalous profiles, far from the simple sigmoid model. Although in such cases formal treatments are generally disregarded, this fact has promoted suspicion about the general validity of the classic DR theory. Before renouncing this conceptual frame, however, it seems more prudent to obey the parsimony principle and to attempt interpretations in accordance with the simple and accepted basis of the theory.

A biphasic response is an interesting anomaly, having two graphical branches with different signs, typically stimulatory at low doses and inhibitory at high doses. This response, which Southam and Ehrlich [1] called 'hormetic', has seen a renewed interest in recent years [2-4], which has led to talk of the 'rebirth of hormesis as a central pillar of toxicology' [5] and has even produced a re-launching document, signed by 58 investigators [6]. In this context, it has been pointed out-with good reason-that the dogmatism of classic toxicology has hindered the recognition of the phenomenon [6-8], as well as its generality [9,10]. Furthermore, it has been suggested that this generality could lead to revision of the environmental protection policies, which are perhaps unnecessarily expensive [4,11,12], and it has also been pointed out that hormesis could lend a conceptual basis to the practice of homoeopathy [13].

In a previous work [14] we have discussed some of these viewpoints and presented theoretical and experimental evidence showing that hormetic responses-at least some of them-could be the result of the simultaneous action of two effectors, treated and interpreted under the hypothesis of a single effector. The bioassay of complex solutions (tissue extracts, biological fluids, cell-free media from microbial cultures, environmental samples and urban and industrial wastes) is, in fact, an experimental context which favours this type of result. Regarding this, studies that allude to hormesis-primarily the pioneering work of Southam and Ehrlich [1]-often come from that experimental context, and the insistence in homoeopathy on the use of "natural" extracts (i.e. without purifying) leads to similar situations.

The presents work examines another source of anomalous DR responses, even to a single effector, related to the population dynamics of the target organism. The first group of experimental results analysed herein was obtained by studying a time-course of the response to two antimicrobial peptides (nisin and pediocin bacteriocins) by *L. mesenteroides *and *C. piscicola *respectively (the first is a bacteria commonly used as an indicator in the bioassay of bacteriocins and the second is a common parasite of fish. The second group of experiments was carried out for comparison and involved a classic antiseptic, phenol, against the same microorganisms.

In three of the six cases studied, we detected different types of anomalous profiles, only some of which can be classified as hormesis. All, however, can be formally described in the frame of the classic DR theory, treated in the dynamic terms that we propose here. These terms facilitate the distinction between genuinely hormetic phenomena and other situations able to generate similar biphasic DR profiles. Finally, from a practical point of view, the results suggest that we should be cautious about use of bacteriocins as antimicrobials in the preservation of foodstuffs.

## Results

**Figure 1:**
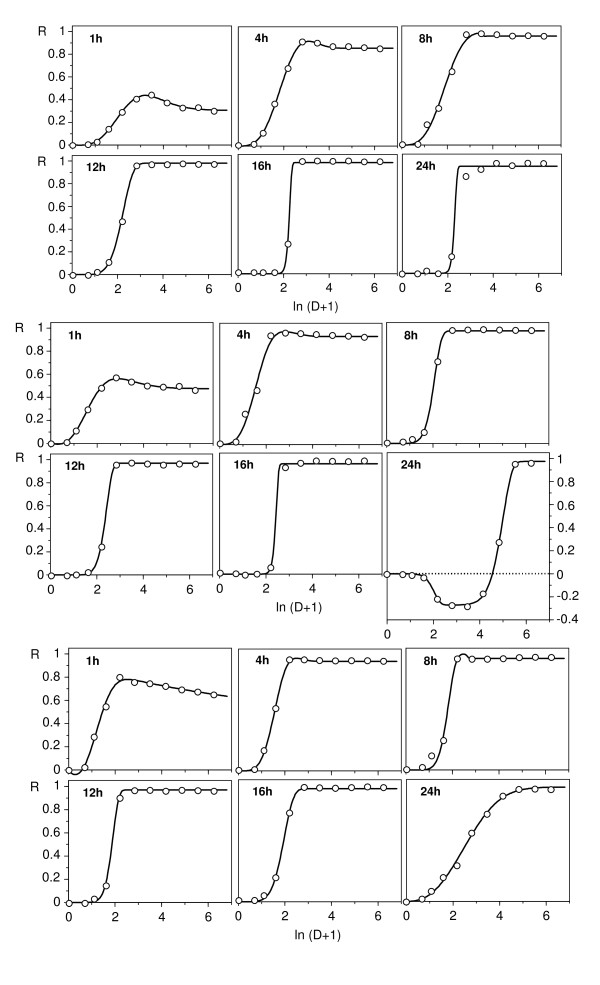
**Response of *L. mesenteroides *to nisin**. Graphic representation of *L. mesenteroides *inhibition growth (R) to nisin (D: dose in mg/l) at different temperatures (from top to bottom: 23, 30, 37°C) and specified exposure times. Experimental results (points) and fittings (lines) to the models (A1) or (A2). For clarity, doses are represented in logarithmic scale, and confidence intervals (in all the cases less than 5% of the experimental mean value; α = 0.05; n = 4) are omitted.

**Figure 2:**
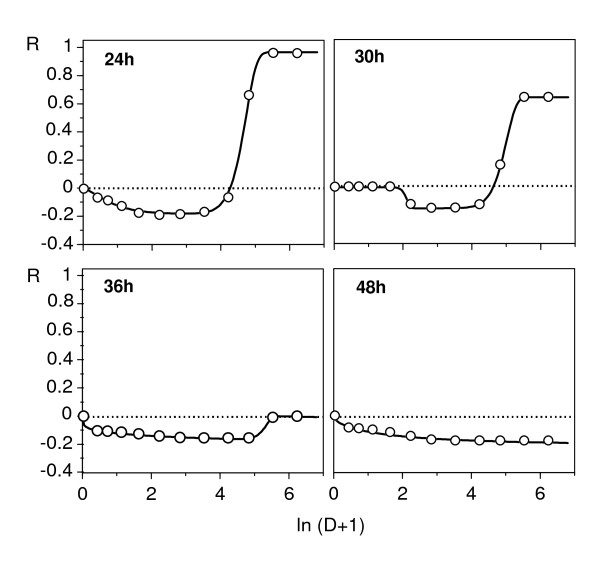
**Response of *L. mesenteroides *to nisin at 30°C and long exposure times**. Graphic representation of *L. mesenteroides *inhibition to nisin at 30°C and long time-course. Experimental results (points) were fitted (lines) to model (A2). Other conventions as in Figure 1.

**Figure 3:**
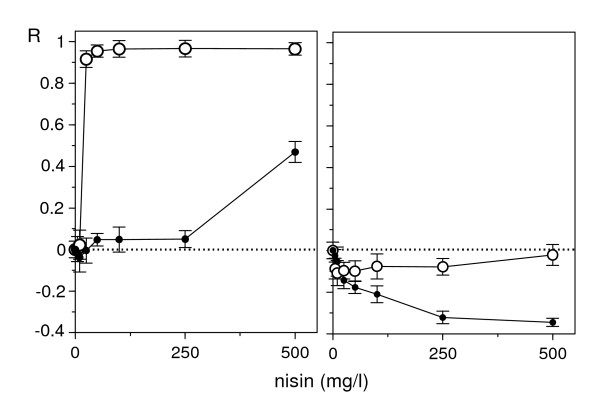
**Responses to nisin of non-habituated and nisin-habituated *L. mesenteroides***. These graphs show responses to nisin non-habituated (white circle) and nisin-habituated (black circle) bacteria at exposure times of 12 (left) and 48 h (right). Error bars indicate confidence intervals (α = 0.05; n = 4). Lines are in this case only indicative, and they do not translate fittings to a specific model.

**Figure 4:**
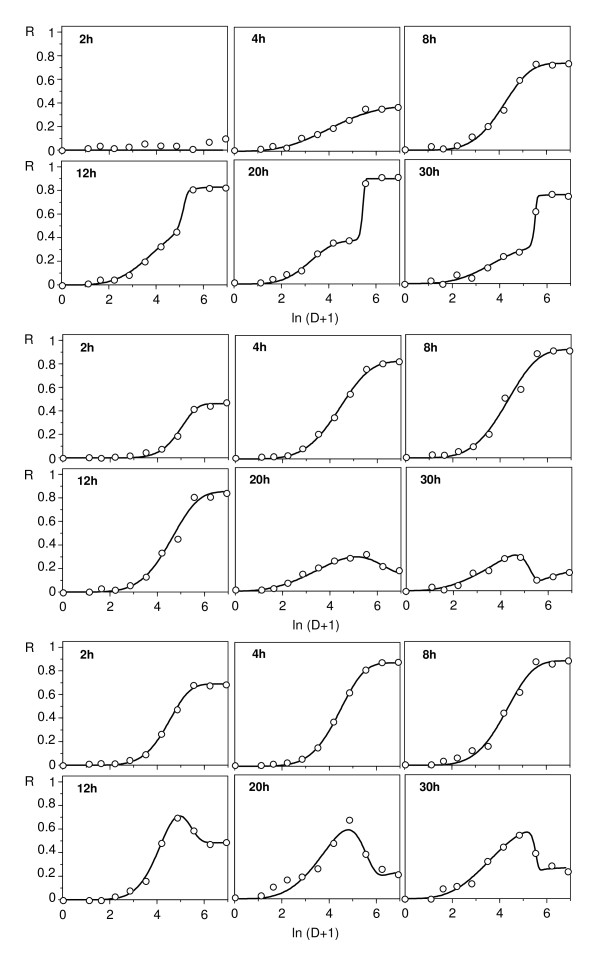
**Response of *C. piscicola *to pediocin**. Graphic representation of *C. piscicola *response to pediocin at different temperatures (from top to bottom: 23, 30, 37°C) and specified exposure times. Experimental results (points) and fittings (lines) to equations (A1) or (A2). Other conventions as in Figure 1.

Figures 1, 2, 3 and 4 show the responses of *L. mesenteroides *and *C. piscicola *to nisin and pediocin respectively, in a wide dose domain, at different temperatures and times (although we tested 10 exposure times, these Figures only show 6 representative cases to avoid redundancies). Furthermore, examples of growth kinetics using data of nisin effect on *L. mesenteroides *at three temperatures are depicted in Additional file 1. Despite the apparent heterogeneity of the DR profiles detected (Figure 1, Figure 2, Figure 3 and Figure 4), the results showed several interesting regularities:

1. An important proportion of profiles deviated from the simple sigmoid equation, which, in the absence of other evidences, could be considered acceptable in some cases. However, moderate and pronounced deviations (in the form of biphasic responses) did not appear randomly, but in time sequences affected by temperature, indicating that these sequences are characteristic of the studied responses. The individual fittings to additive models (see Appendix and Table 1 for parameter definitions) were in all cases statistically significant in their parameters (Student's t; α = 0.05) and consistent in their form (Fisher's F; α = 0.05).

**Table 1:** Symbolic notations used and corresponding units

	
*Weibull equation (original and reparameterized forms)*	

*R:*	Response as inhibition of bacterial growth. Dimensionless
*D:*	Dose. Dimensions: mg/l
*b:*	Position parameter. Dimensions: mg/l
*a:*	Shape parameter. Dimensionless
*m:*	Dose for semi-maximum response (ED_50_). Dimensions: mg/l
*K:*	Maximum inhibition response. Dimensionless

*Logistic equation and biomass dynamic*	

*X:*	Biomass. Dimensions: mg/l
*t:*	Time. Dimensions: h
*v_x_:*	Biomass production rate. Dimensions: mg l^-1 ^h^-1^
*X_m_:*	Maximum biomass. Dimensions: mg/l
*r_0_:*	Specific maximum rate without effector action. Dimensions: h^-1^
*r:*	Specific maximum rate with effector action. Dimensions: h^-1^
*Q_0_:*	Initial effector concentration. Dimensions: mg/l
*Q_H_:*	Concentration of effector retained by dead biomass (*X_H_*). Dimensions: mg/l
*q_H_:*	First order kinetic constant. Dimensionless
*v_Q_:*	Rate of available effect dynamic. Dimensions: mg l^-1 ^h^-1^
*Q_S_:*	Concentration of effector metabolically deactivated by living biomass (*X_S_*). Dimensions: mg/l
*q_S_:*	Second order kinetic constant. Dimensions: l mg^-1 ^h^-1^
D*:	Dose:Biomass ratio. Dimensionless

*Subscript meaning*	

*H:*	Death
*S:*	Survival
*m:*	Maximum

2. The time-course of the response included an initial period with increasing asymptotic values of the inhibitory effect, followed by the progressive accentuation of a biphasic response. In nisin, the first experimental series showed a sole case (24 h at 30°C; Figure 1) of biphasic response with a stimulatory branch at low doses. Additional assays at longer times (Figure 2) confirmed this result and showed that the stimulatory branch involved progressively increasing nisin levels, until reaching the whole domain of the dose at 48 h.

This behaviour suggested that a fraction of the bacterial population was stimulated by nisin, or it developed this ability during the exposure time, thus prevailing gradually on the inhibited fraction. To verify this hypothesis, an inoculum of the microorganism was incubated under the bioassay conditions in the presence of 250 mg/l nisin and, after 48 h, an aliquot of the population was subjected to a repetition of the same treatment. Immediately, new DR tests were carried out to compare the responses at 12 and 48 h of the nisin-habituated population and a non-habituated inoculum. The results (Figure 3) showed that in the habituated population the inhibitory effect at 12 h was significantly lower than in the non-habituated one, whereas at 48 h the stimulatory effect was significantly higher.

3. In initial stages, the increase of temperature in the 23-37°C interval accelerated the response, reducing the time necessary to reach maximum inhibition, but scarcely altering the value of this inhibition. Thus, the absolute maxima with pediocin at 23, 30 and 37°C were reached at 20, 8 and 6 h, with very close inhibition values (asymptotes at 87.5, 91.5 and 90.4%, Figure 4). The response of *L. mesenteroides *to nisin was similar, although with a quicker development and a more intense inhibition. This suggested, therefore, that the temperature affects the rate of the processes responsible for toxicity, but does not alter the factors which determine them; that is, the affinity of the receptors by the effector is increased, but the number of receptors cannot be increased. At the last stage, the response accelerated in the 23-30°C interval and was delayed in the 30-37°C interval (with a more pronounced biphasic response of *L. mesenteroides *to pediocin).

In these conditions, the usual description of the DR relationships at an arbitrary exposure time is not very satisfactory, since different times yield very different conclusions. The response to nisin at 30°C, for example, could be classified as inhibitory (up to 24 h), hormetic (24-48 h) or stimulatory (more than 48 h). The case of pediocin appears to be even more complex, because the biphasic profiles in the second stage even seem to produce a hormetic response.

**Figure 5:**
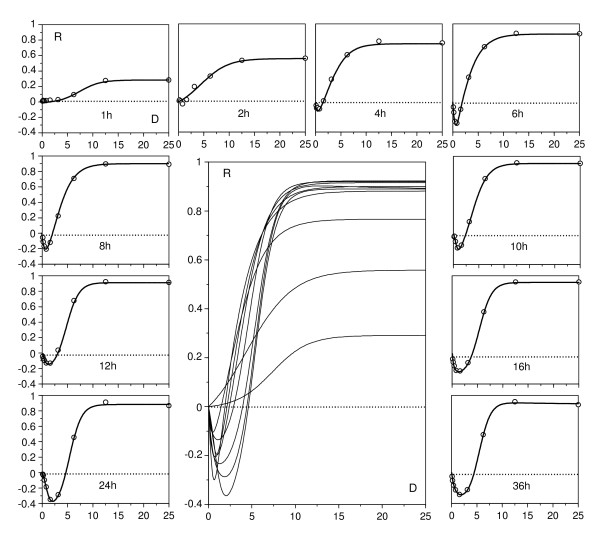
**Time-course of the response of *C. piscicola *to phenol**. These experimental results (points) were fitted (lines) to equation (A2). The phenol concentrations (D) were given in g/l. The central graph -which collects all the results, omitting experimental points-allows to detect the restriction of the stimulatory response (negative R) throughout the time to a small domain of low doses.

With the aim of obtaining data about the response of the same microorganisms to other antimicrobial agents, the same type of bioassay was applied using penicillin and phenol, with sampling throughout an exposure period of 36 h. In three of these four cases, inhibitory conventional responses (not shown) were detected. However, in *C. piscicola*, phenol yielded a more defined stimulatory branch at low doses (Figure 5), and, unlike nisin, the dose interval corresponding to this stimulatory effect remained essentially constant throughout the bioassay period.

## Discussion

Setting the hormetic hypothesis aside for the moment, we know that a possible cause of the biphasic profiles is the simultaneous action of two effectors [14,15]. We previously pointed out that the (frequent) testing of complex solutions is a favourable context for biphasic responses, but a single effector can also produce them, because even a very simple molecule can split into multiple forms with different affinities for the receptor (for example, an ionic species and another covalent in equilibrium depending on pH). Thus, lactic acid is toxic to many organisms in its covalent form but not in its ionic state [16,17]. Therefore, we only need to suppose that the ionic form promotes a stimulatory response (or simply that the target organism can use the lactate as a nutrient), to obtain a profile which decreases after reaching its maximum.

The cases described here, however, seem to be of a different nature, and they suggest the coexistence of two different types of response in the populations studied. The results shown in Figure 3 indicate that the exposure to nisin produces an enrichment of the initial microbial population in a subpopulation with stimulatory response, without disappearance (at least up to 250 mg/l of nisin) of the subpopulation with inhibitory response. We can conclude that under the bioassay conditions, at least during a large extent of the exposure time, two subpopulations with different sensitivity to nisin coexist, which is equivalent to a population with a bimodal distribution of sensitivity to this peptide.

The kinetic approach applied here can neither certainly establish the mechanism of action nor define the nature of the chemical species potentially involved in the detected effects. Therefore, what interests us now is to determine if the DR theory, combined with the basic hypothesis of the microbial population dynamics, is sufficient to explain the detected variety of profiles.

### A dynamic DR model

In a DR assay involving microorganisms or cell populations with a high renovation rate, the exposure period could include various generations of the biological entity. It approaches the problem to the case of the chronic toxicity, from which it differs because there is no constant intake of the effector into the system. In such a case, the classic DR models can be insufficient, as they omit the kinetic perspective. For example, consider the state of a population subjected to sublethal effects, containing effector-immune elements or able to develop detoxifying resources during such a time. Under these conditions, a more realistic model arises from the following set of hypotheses.

#### A. Hypothesis concerning the action of the effector

A1. The effector can determine the drop of the living biomass (*X*) due to cell death, or the drop of the maximum specific growth rate (*r*). In both cases we admit that the response R can be described by means of model A1 (see Appendix and Table 1 for parametric definitions and units), where the subindex *φ *can take the values *X *and *r *according to the specific response considered:(1)

A2. In accordance with the usual convention of a total biomass *X*, when *X_H _*dies at a given dose of the effector (*X_S _*being the surviving biomass), the response *R_X _*in terms of biomass will be:(2)

A3. Similarly, if the response *R_r _*in terms of the maximum specific rate is a decrease from *r_0 _*to *r *in the absence of the effector, we will have:(3)

The adequate formulations for an effector with stimulatory action (response with negative sign, see methodological section) are obtained in a similar way. Since the increase in cell number can only be attributed to the (-)*R_r _*response, the meaning of the (-)*R*_X _response is the increase of dry weight per cell. Thus, when biomass is estimated by means of absorbances or number of colony forming units, it is only pertinent to consider the response in terms of maximum specific rate.

A4. Bearing in mind the preceding specification, if a total biomass *X *increases up to a value *X_S _*(where *X_S _*= *X *+*ΔX*) at a given dose of effector, the response will be:(4)

A5. Similarly, if the response *R_r _*of the maximum specific rate is the increase to a value *r *from a value *r*_0 _in the absence of the effector (with *r *= *r_0 _*+ *Δr*), we will have:(5)

#### B. Hypothesis concerning biomass dynamics

We accept that the biomass *X *grows according to a conventional logistic equation, whose differential expression is [18]:(6)

where *r*_0 _is the maximum specific growth rate in the absence of the effector, and *X_m _*is the maximum biomass. In the presence of the effector, the constant *r*_0 _turns into the variable *r *(which is dependent on the dose); therefore, this differential form cannot allow an analytic solution. Therefore, the expression (6) will be directly used later on in the numeric solution of the system.

#### C. Optional hypothesis concerning the dose

The dose *D *is commonly considered a constant: it is the initial concentration of the effector, which is a good criterion when the biomass does not vary appreciably during the exposure time. However, this approach can be doubtful if the action of the effector reduces (without cancelling) the growth rate, because in this case the ratio of available effector to biomass diminishes with time. Indeed, it is difficult to accept that in a microbial culture the initial level of effector means the same against the initial biomass as against a biomass often larger by several orders of magnitude a few hours later. In fact, these considerations are implicit when a clearly specified value of the initial biomass is required for standardizing DR assays.

This problem seems especially relevant if the dose is low with regard to the initial biomass, and the affinity between effector and biomass is high. Thus, for example, in 0.25×10^6 ^cells/ml suspensions of the marine diatom *Thalassiosira rotula *in a medium with 200 ng/ml of Arochlor-1248 (a formulation of polychlorinated biphenyls), the biomass concentrated in 60-120 minutes approximately 45% of Arochlor, what meant 90% of the available one, since other 45% was adsorbed on glass walls and 5% remained in the medium [19]. It is known that lipophilic compounds can be concentrated very quickly by the biomass through hydrophobic repulsion, partition and adsorption mechanisms, but the phenomenon is not necessarily restricted to these processes.

Under such conditions, the dose could probably be defined more appropriately as the ratio of total initial effector *Q*_0 _to the present biomass:(7)

It can also be pertinent to admit that a part *Q_H _*of the total initial quantity *Q_0 _*of effector is retained by the dead biomass, and another part *Q_S _*is metabolically deactivated by the living biomass. The simplest hypothesis consists of accepting that the quantity *Q_H _*is proportional to the dead biomass:(8)

while *Q_S _*is formed through a second order kinetic equation (first in each component), at a rate *v_Q _*dependent on the concentrations (or quantities in constant volume systems) of living biomass and available effector (*X_S _*and *Q*):(9)

The first supposition can be suitable with effectors that form covalent bonds with the receptor, or that have a hydrophobic character and tend to be concentrated by the biomass, as we said before. The second can be applicable to effectors which are transformed into inactive metabolites, or chemical species whose action can be modelled by means of sets of equations (1) to (5). If such suppositions are necessary, dose could be defined as:(10)

Whichever definition of dose we establish, hypotheses A1-A5 allow us to determine the biomass at a time instant *t *as a function of the biomass at (*t*-*Δt*) by means of the following balance (supposing an effector that reduces cell viability and growth rate):(11)

where ^m^W_φ,D _are the responses to the dose *D*, in terms of cell death or *r *drop, according to equation (1). If the effector is stimulatory in the sense defined in A4 and A5, the signs of the terms ^m^W_φ,D _should be changed.

### Results from the dynamic model

Using biologically reasonable parametric values and a small time increment (*e.g*. Δ*t *= 0.005) to minimise the error of the differential approximation, equation (11) allows us to simulate response surfaces as a simultaneous function of dose and time, for different assumptions about the growth and the action of the effector. Without loss of generality we can simplify and disregard the options (8) to (10), that is, we can suppose *q_H _*= 0 and *q_S _*= 0. Under these conditions it is suitable to distinguish three categories of facts:

S1. The effector can depress the cell viability (*X*-action) or the specific growth rate (*r*-action); the joint effect is a trivial result of the separate effects.

S2. The dose can be considered constant and equal to the initial concentration of effector, or variable according to equation (7). We will call these cases D_cst _and D_var _respectively.

S3. The population distribution of the sensitivity to the effector can be uni- or bimodal, with notations P_uni _and P_bi _respectively. The second case-equivalent to two subpopulations with different sensitivity-is obtained by applying equation (11) to two populations with different parametric definitions and calculating the response on the sum.

**Figure 6:**
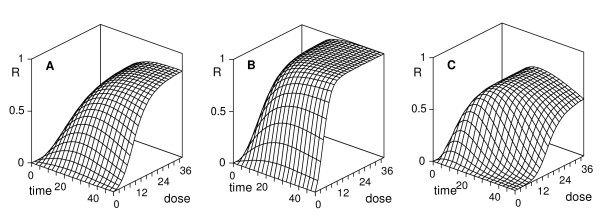
**Response surfaces as simultaneous functions of dose and time**. Simulations performed by means of the dynamic model (11), under the hypothesis about the action of the effector, sensitivity of the target microbial population and dose metrics specified in Table 2.

**Figure 7:**
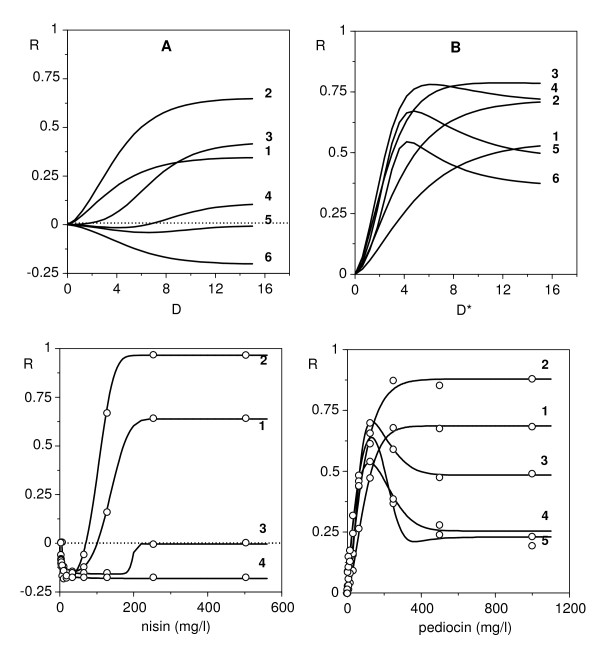
**Theoretical simulations and mathematical fittings of the toxico-dynamic model**. Up: two simulations (A and B) of the time series of responses generated by means of the dynamic model (11) under the conditions specified in Table 2. Down: real time series corresponding to the cases of nisin at 30°C (Figure 2) and pediocin at 37°C (Figure 4), here treated in natural values to facilitate comparison. Graph superscriptions indicate time sequences.

With P_uni _populations (Figure 6, parameters in Table 2), the DR profile can always be fitted to a simple sigmoidal model, though the time profile depends on other factors. In *X*-actions, the asymptote of the response ascends progressively with time until a maximum and constant value. In *r*-actions, the asymptote of the response ascends to a maximum and then drops, more markedly in D_var _than in D_cst_. More interesting are the P_bi _populations, especially when the effector inhibits a subpopulation and stimulates the other one. Figure 7 (parameters in Table 2) shows two simulations of this hypothesis and demonstrates that model (11) allows us to generate all the types of biphasic profiles detected in the above described bacteriocin assays.

**Table 2:** Parameters from equation (11) used in the simulations of Figures 6 and 7

	growth model			DR_X _model			DR_r _model		
cases		pop 1 **^a^**	pop 2 **^a^**		pop 1	pop 2		pop 1	pop 2

fig 6A	*X_0_*	0.100	-	*K_X_*	-	-	*K_r_*	0.900	-
	*r_0_*	0.100	-	*m_X_*	-	-	*m_r_*	10.000	-
	*X_m_*	1.000	-	*a_X_*	-	-	*a_r_*	1.500	-

fig 6B	*X_0_*	0.100	-	*K_X_*	0.001	-	*K_r_*	-	-
	*r_0_*	0.100	-	*m_X_*	10.000	-	*m_r_*	-	-
	*X_m_*	1.000	-	*a_X_*	1.500	-	*a_r_*	-	-

fig 6C	*X_0_*	0.150	-	*K_X_*	-	-	*K_r_*	0.800	-
	*r_0_*	0.150	-	*m_X_*	-	-	*m_r_*	30.000	-
	*X_m_*	1.000	-	*a_X_*	-	-	*a_r_*	1.500	-

fig 7A	*X_0_*	0.050	0.050	*K_X_*	-	-	*K_r_*	0.600	1.000 **^S^**
	*r_0_*	0.500	0.025	*m_X_*	-	-	*m_r_*	4.000	4.000 **^S^**
	*X_m_*	1.000	1.000	*a_X_*	-	-	*a_r_*	1.500	1.500 **^S^**

fig 7B	*X_0_*	0.200	0.050	*K_X_*	0.002	-	*K_r_*	0.600	1.000 **^S^**
	*r_0_*	0.150	0.050	*m_X_*	4.000	-	*m_r_*	3.000	4.000 **^S^**
	*X_m_*	1.000	1.000	*a_X_*	1.500	-	*a_r_*	1.500	1.500 **^S^**

In 6C, the dose is considered as the ratio of initial effector level to biomass in each time instant. In the rest of the cases the habitual criterion is applied (dose as initial concentration of the effector).

(**^a^**): populations 1 and 2, as defined by means of the parameters of growth and DR models.

(**^S^**): parameters corresponding to stimulatory responses.

**Figure 8:**
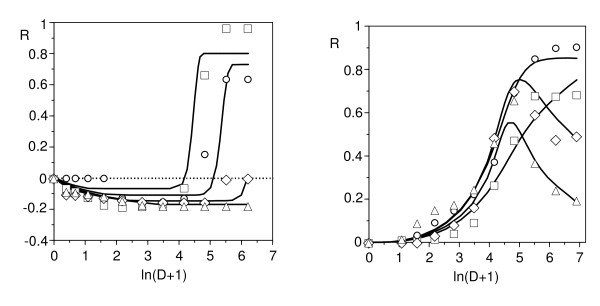
**Experimental biphasic responses of *L. mesenteroides *fitted to the toxico-dynamic model**. The dynamic model (11) was utilized as a solution for two especially complex time series of responses in *L. mesenteroides*. Left: against nisin, at 30°C (square: 24, circle: 30, rhombus: 36, triangle: 48 h; see Figure 2); right: against pediocin, at 37°C (square: 2, circle: 8, rhombus: 12, triangle: 20 h; see Figure 4).

Finally, equation (11) was tested as a simultaneous solution for the time-course series of the responses in two representative cases: nisin against *L. mesenteroides *at 30°C (Figure 2), and pediocin (2, 6, 12 and 20 h) against *C. piscicola *at 37°C (Figure 3). Fittings were reasonable in both cases (*r*^2 ^= 0.964 and 0.985 respectively, Figure 8), and their results, although not accurate in the details, were consistent with the simulations of the Figure 7. They described satisfactorily the essential and most notable character of the responses, that is, the gradual transitions among inhibitory, stimulatory and biphasic profiles. It is interesting to point out that the best fit was obtained under the D_var _hypothesis in the first case and D_cst _in the second. This result suggests, beyond its literal interpretation, the existence of differences in the processes acting on the effector throughout the exposure period. Thus, the excessive schematism of model (11), among other reasons to avoid too many parameters, is possibly a cause of the above mentioned inaccuracy.

Equation (11) can be now considered under two perspectives. First, as a description of reality, it cannot guarantee-as it happens in any kinetic model-the validity of the interpretation which it proposes, in this case the existence of two subpopulations. Regarding this, however, the results depicted in Figure 4 indicate that an exposure time of 48 h to pediocin promotes a change in the proportions of cells that respond in a different way to the peptide. This leads us to conclude that two subpopulations are present, at least at this time point. Under a complementary perspective, equation (11) is only a valid combination of two well-validated descriptions: the kinetic model of microbial growth in a limited medium, and the probabilistic model of DR relationships. Thus, any simulation derived from such a combination is a (perhaps unexpected) result that will arise in reality whenever a tested population includes two subpopulations with the characteristics provided by the specified parametric values.

### The hormetic response

As characterised by Southam and Ehrlich [1], hormesis is «a stimulatory effect of subinhibitory concentrations of any toxic substance on any organism». The typical manifestation of this phenomenon is a biphasic response with two branches of opposite sign: stimulatory at low doses and inhibitory at high doses, but the reciprocal statement is not advisable. A biphasic response necessarily reveals the combination of two different phenomena, and so it can take place when two effectors act on a population with unimodal sensitivity [14,15], or, as in the cases studied here, when a single effector acts on a population with bimodal sensitivity. However, none of these cases has connection with the *sensu stricto *hormesis, which implies a duality of mechanism. Since the current rebirth of interest in this phenomenon can lead to supposing a hormetic response instead of a biphasic response from other origins, it seems opportune to emphasise that the definition of hormesis cannot be limited to the biphasic character of the response, but it should imply two conditions:

C1. A single effector acts on a population with unimodal distribution of the sensitivity, through two mechanisms, each affecting a different subsystem of the target organism.

C2. Both mechanisms exert effects of opposite sign on the global variable which is used to quantify the response.

This response will be able to be described by means of a degenerate biphasic subtractive model (see Appendix), in which the parametric values of *K *and *m *are lower in the stimulatory term than in the inhibitory one. But beyond the problem of the formal description, two questions arise: the first refers to the realism of conditions C1 and C2; the second refers to possible criteria to distinguish a strictly hormetic response from biphasic responses due to other factors.

The condition C1 is realistic: vitamin A damages the retina if it is deficient and the liver when it is in excess [20]. Actually, the sign inversion of the response is accepted as an almost trivial fact when the depressor effect is derived from the excess of a stimulatory effector: thus, a nutrient like sucrose inhibits microbial growth at concentrations that are able to significantly reduce the water activity, a phenomenon that is the basis of marmalades. The opposite fact (a toxin that has a favourable effect at low doses) is simply less intuitive and more difficult to detect and use practically, but not necessarily less probable. The condition C2-the existence of variables that can translate the combination of two modes of action-seems more problematic. However, many effectors induce the synthesis of detoxifying enzymes with a low specificity. These can act on endogenous substrates and activate mechanisms of stimulatory meaning (electronic transport, production of biologically active metabolites, hydroxylation of steroid hormones, cell division) that predominate at low doses and are counteracted by the principal action of the effector at higher doses.

The second question (distinguishing between hormetic and biphasic responses) raises the same problem discussed in connection with equation (11). Indeed, to state strictly that a certain response is hormetic requires identification of the mechanisms that determine it. However, the results discussed as far suggest that important toxicodynamic evidence in favour of a hormetic hypothesis could be the essential constant throughout the time of the relationship between the two phases of the response. In other words, if there is a single effector and there are no subpopulations with different sensitivities, the relative length of the two branches of the response only depends on dosage, not on time, which impedes the progressive predominance of one branch over the other, as can be seen in the response to nisin (Figure 2).

It is difficult to specify *a priori *the characteristics of an effector able to produce a hormetic response in a given organism. Thus, phenol was selected for comparison because three features suggest its adequacy for this purpose: 1) it can be considered a single effector, as the weakly acidic character of its hydroxylic hydrogen makes only a negligible proportion of the ionic form in the assay conditions; 2) it is a well known, vigorous and not very specific antiseptic; 3) phenols are obligatory steps in the biodegradation of the aromatic hydrocarbons, a process which is initiated in many organisms by an active enzyme induction with a detoxifying role. The response obtained with *C. piscicola *(Figure 5), a stable stimulatory branch at low doses that did not progress over time at the expense of the inhibitory branch, is solid evidence in favour of a hormetic phenomenon.

## Conclusions

The responses of *L. mesenteroides *to nisin and *C. piscicola *to pediocin showed variation over time, which generated anomalous DR profiles far from the simple sigmoid model. Some of these profiles were of the biphasic type with two branches of opposite sign, a characteristic that is usually attributed to a hormetic phenomenon.

Our results show, however, that the combination of the kinetic model of microbial growth and the probabilistic model of DR relationships can generate time series with very different profiles, including all the anomalies detected in practice. In a complementary way, the dynamic model developed satisfactorily fits the most remarkable trends of the experimental time succession of responses, when we accept that the microbial populations assayed contain-or develop during the exposure time-subpopulations with different sensitivity to bacteriocins.

Therefore, although the biphasic profiles can be derived from a genuinely hormetic response, they can also arise when two effectors act on a bimodal-sensitive population [14,15], or, as in the cases studied here, when a single effector acts on a unimodal-sensitive population. Any of these suppositions can be accurately described by means of a subtractive degenerate model (see Appendix), but to distinguish among them requires identification of the underlying mechanism. Toxicodynamic evidence in favour of the hormetic hypothesis could be the stability in the time of the dose intervals which define the two branches of the curve, as in the response of *C. piscicola *to phenol.

Additional consequences of these results follow. 1) The need to verify the kinetics of the response and the presence of a single effector before deciding that we are looking at a case of hormesis. In a previous work [21], we demonstrate that the response is a sigmoidal function of time for the same reasons for which it is a sigmoidal function of dose (the most sensitive elements of the population not only respond at lower doses but also at shorter times). Therefore, the examination of the time-course of the response, in any case with a well defined toxicological interest, is especially important if anomalies are detected in an assay at only one exposure time. 2) The inadequacy of the plate assays based on inhibition zones. These are qualitatively useful, but too imprecise to detect the effects mentioned here. 3) The need to confirm carefully the antimicrobial effects of the bacteriocins in the specific conditions of their application, when they are used as agents for the control of undesirable microbiota in food products.

## Methods

### Reagents

The tested agents were nisin, phenol (both from SIGMA) and pediocin. The last was prepared from a *Pediococcus acidilactici *NRRL B-5627 culture in MRS medium, according to the process described by Vázquez *et al*. [22].

### Microorganisms and bioassay

The microorganisms used were *Carnobacterium piscicola *CECT 4020 and *Leuconostoc mesenteroides *subsp. *lysis *(kindly provided by Dr. Ray, University of Wyoming, Laramie, USA), both commonly used as indicators in bacteriocin bioassays. Experiments were carried out in quadruplicate, using methods which were described in detail in previous studies [23-25].

To prepare the microbial suspensions, cultures aged 12 h in MRS medium were centrifuged, the sediment washed with 0.05 M, pH = 6.0 biphtalate-NaOH buffer in fresh MRS medium (MRS-f), and the washed sediment resuspended in MRS-f and adjusted to an absorbance (700 nm) of 0.200. For DR analysis, four series of dilutions in MRS-f were prepared with each effector, and the assay began combining equal volumes (1 ml) of microbial suspension and effector solution (MRS-f in the control). Incubations were performed in 15 ml tubes at 23, 30 and 37°C, with 200 rpm orbital shaking, and the results were quantified as *R *= 1-(*A*_D_/*A*_0_), where *A*_0 _and *A*_D _are the absorbances at 700 nm of the control and the dose *D *respectively. The inhibitory and stimulatory responses have thus positive and negative sign, respectively.

For comparative purposes, *A*_D _and *A*_0 _quantifications were performed in some cases by plate count on MRS-agar with similar results to those obtained from absorbances (data not shown). However, attempts to carry out systematic inhibition bioassays by means of the usual plate method of the clear zones (halos) produced qualitatively similar, but more inaccurate results.

### Numerical methods

Fitting procedures and parametric estimations from the experimental results were performed by minimisation of the sum of quadratic differences between observed and model-predicted values, using the nonlinear least-squares (quasi-Newton) method provided by the macro '*Solver*' of the *Microsoft Excel XP *spreadsheet. Subsequently, confidence intervals from the parametric estimations (Student's *t *test) and consistence of mathematical models (Fisher's *F *test) were determined using *DataFit 9 *(Oakdale Engineering, Oakdale, PA, USA).

## Competing interests

The authors declare that they have no competing interests.

## Authors' contributions

Both authors contributed equally to this work. MAM and JAV provided the information to construct the mathematical models, performed all the microbiological experiments and data analysis and they wrote the manuscript. Both authors read and approved the final paper.

## Appendix. Dr Models Used

### Simple sigmoid response

In previous works [14,21,23,26], we have discussed in detail several general problems of the DR modelling, and we have proven the fitness of the cumulative function of the Weibull distribution. Its use as a DR model requires two modifications: 1) we multiply the second member by the maximum response *K*, so that the asymptote can take values lower than 1, and 2) we reparameterized the equation, so that it explicitly includes the dose for semi-maximum response (ED_50_, *m *in our notation). This facilitates the test of initial values in nonlinear fitting methods, and allows the direct calculation of the parametric confidence intervals by means of the usual software. The final form, which we will denote ^m^W, is:(A1)

where *D *is the dose, *R *the response (with *K *as asymptotic maximum), *m *the dose for semi-maximum response and *a *the form parameter related to the maximum slope of the response.

### Biphasic profiles and degenerate additive responses

**Figure 9:**
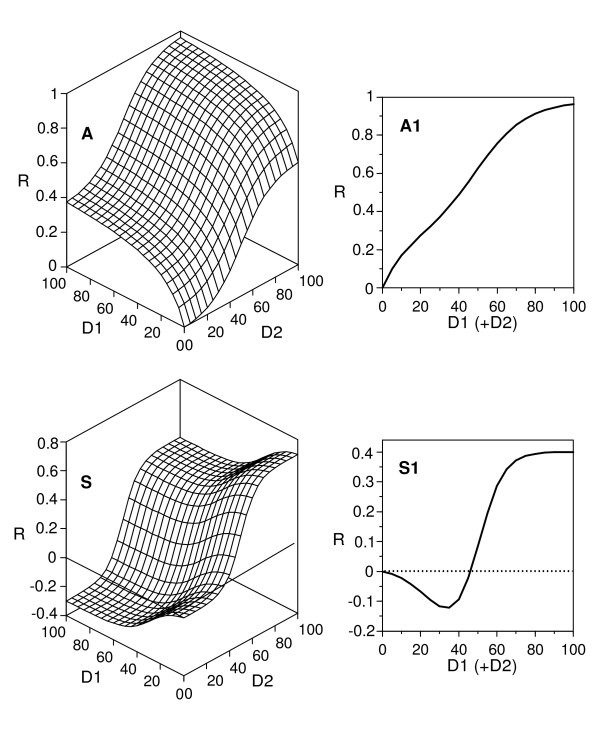
**Simulations of responses to the simultaneous action of two effectors**. These simulations were generated by means of the model (A2) and were additive (A) and subtractive (S) responses to the joint effect of two agents. Right: degenerate responses which are obtained when treating the results as a function of a series of dilutions from a solution containing both effectors.

The bioassay of complex solutions (tissue extracts, biological fluids, cell-free media from microbial cultures, environmental samples and urban and industrial wastes) can produce several types of biphasic responses. Although often they are attributed to hormesis, they can be explained easily in terms of a model of additive effects (different from the habitual concentration addition and independent action hypotheses), with loss of one independent variable. Indeed, consider the assay of a solution containing two effectors whose actions imply additive effects. In such a case, a rigorous description of the response would require a bivariate function (two doses; Figure 9, left) of the type:(A2)

However, if the response is simply expressed as a function of the dilution, a common practice in the preliminary examination of materials as those above mentioned, or if one only bears in mind a sole effector, the result is equivalent to what would be obtained selecting the values of the response on the line bisecting the plane defined by the two independent variables (Figure 9, right).

If both responses imply the same values for *m *and *a*, the profile will be able to be described by means of a simple sigmoidal model (^m^W). In another case, the profile will be biphasic and its description will require the same model (A2) appropriate for the bivariate description, using now the same independent variable (*i.e*., dilution) in both additive terms. The fit will be satisfactory, but the parametric estimates thus obtained will only represent a combination of the responses due to the correlation of increasing doses of the two effectors. In the case of two effectors with effects of opposite sign, the profile will show features of hormesis, and the appropriate model will be subtractive (Figure 9S). A similar analysis is applicable to the case of a single effector against a population with a bimodal distribution of sensitivity.

On the other hand, if the number of effectors (or the number of subpopulations with different sensitivity to a single effector) increases, the overlap of the different responses tends to smooth the waves of the profile. Under these conditions, such waves are easily absorbed by the experimental error, and the result can be fitted again to a simple sigmoidal model.

## Supplementary Material

Additional file 1 **Figure A1: Effect of nisin on *L. mesenteroides *growth at three temperatures**. In this Figure the effect of nisin on *L. mesenteroides *growth, measured as absorbances at 700 nm, is shown. The experimental data were done at three temperatures (23°C, 30°C and 37°C). The concentrations of nisin tested were (in mg/l): Control without nisin (white circle); 0.98 (black triangle); 1.95 (black square); 3.90 (black rhombus); 7.80 (black star); 15.60 (white square); 31.25 (white down-triangle); 62.50 (white triangle); 125 (white rhombus); 250 (black circle); 500 (black down-triangle). Click here for file
