# Supplementary material for: Biphasic toxicodynamic features of some antimicrobial agents on microbial growth: a dynamic mathematical model and its implications on hormesis
Source: BMC Microbiol. 2010 Aug 19;10:220. doi: 10.1186/1471-2180-10-220 (PMC2936355; doi:10.1186/1471-2180-10-220)
Supplement: Supplementary file 1 [file 1471-2180-10-220-S1.DOC]

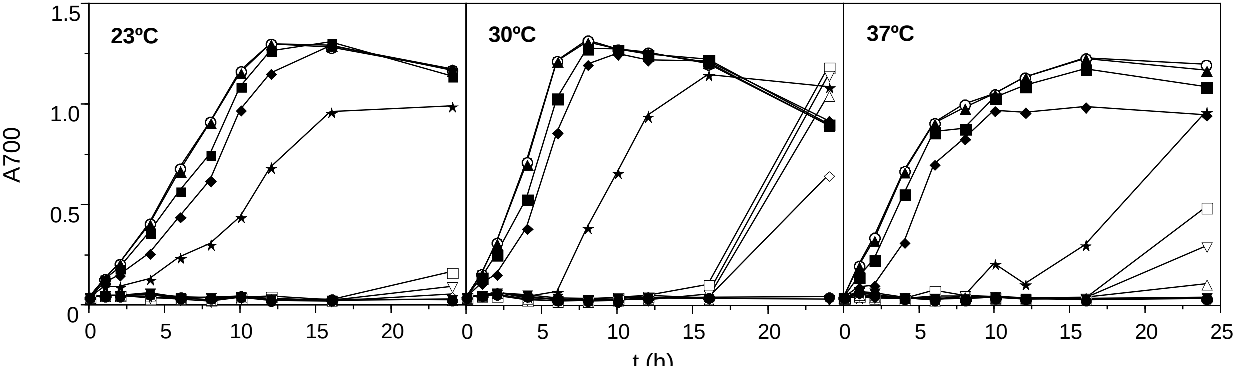

23ºC
30ºC
37ºC
A700
t (h)
0
0.5
1.0
1.5
0
5
10
15
20
25
